# Attendance barriers experienced by female health care workers voluntarily participating in a multi-component health promotion programme at the workplace

**DOI:** 10.1186/s12889-018-6254-3

**Published:** 2018-12-04

**Authors:** Pia Maria Ilvig, Thomas Viskum Gjelstrup Bredahl, Just Bendix Justesen, Dorrie Jones, Jonna Benner Lundgaard, Karen Søgaard, Jeanette Reffstrup Christensen

**Affiliations:** 1 Department of Public Health, University of Southern Denmark, J.B. Winsløws Vej 9A, 5000 Odense C, Denmark; 2 Department of Sports Science and Clinical Biomechanics, University of Southern Denmark, Campusvej 55, 5230 Odense M, Denmark; 3 Department of Public Health, Section for Sports Science, Aarhus University, Dalgas Avenue 4, 8000 Aarhus C, Denmark; 4 Department of Clinical Research, University of Southern Denmark, Winsløwparken 19, 5000 Odense C, Denmark

**Keywords:** Worksite, WHPP, Compliance, Adherence, Maintained effect, Sickness absenteeism, Exercise, Qualitative interviews

## Abstract

**Background:**

Studies have shown that Workplace Health Promoting Programmes (WHPP) can facilitate healthier behaviour. Despite the benefits achieved from participating in a WHPP, a systematic review showed that only 10–50% of the employees participated and a challenge was lack of participation. Previous studies stress that understanding the barriers that prevent participants from attending WHPPs are important for designing highly effective interventions. To exploit the potential of a WHPP, it requires a deep insight into the attendance barriers experienced by the participants who voluntarily sign-up for a WHPP; and particularly those who want to stay in the programme but are prevented from participating in it regularly. Thus, the aim of this study was to identify and explore attendance barriers experienced by female Health Care Workers (HCWs) who voluntarily participated in a weekly one-hour multi-component training session, within a WHPP, over a one-year period.

**Methods:**

This study was carried out within a RCT named FRIDOM (FRamed Intervention to Decrease Occupational Muscle pain) and was designed as a single-case study with an inductive approach for analysing the content of in-depth semi-structured qualitative interviews. Data was collected at two home care workplaces and two retirement homes in Denmark. Nine HCWs from the intervention group were selected as participants in the present study.

**Results:**

The attendance barriers identified, consisted of three main themes and six related sub-themes: 1) *organizational factors* (work inflexibility, lack of support from team leaders), 2) *intervention factors* (training sessions organized outside normal work hours, incongruence between information received and reality, content and intensity of the program) and 3) *individual factors* (personal factors).

**Conclusion:**

Organizational and intervention factors are the two most important attendance barriers in future WHPPs. To overcome these barriers; training sessions should be organized within or in connection with work hours, support should be secured from team management and work shifts should be planned to enable attendance for all participants. Furthermore, the attendance barriers may be minimized by including participants in the decision-making process. This relates to both the content and intensity of the intervention, not only in the planning stage but throughout the intervention process.

**Trial registration:**

ClinicalTrials.gov. NCT02843269 - 06.27.2016 - retrospectively registered.

**Electronic supplementary material:**

The online version of this article (10.1186/s12889-018-6254-3) contains supplementary material, which is available to authorized users.

## Background

The World Health Organization has appointed the workplace as a primary setting for promotion public health in the twenty-first century [1]. Studies have shown that health promoting programmes at the workplace can facilitate healthier behaviour and lifestyle, both at work and at home [2–5]. Despite the benefits achieved from participating in a Workplace Health Promotion Programme (WHPP), a systematic review including 23 studies showed that only 10–50% of the employees in WHPP participated [6]. According to the review, the challenges of implementing a WHPP were lack of participant participation and participants leaving the programme [6]. The studies in the review primarily examined employees who either chose to sign-up for a WHPP or chose not to sign-up [7]. The review did not focus on employees who voluntarily signed-up to participate in a WHPP but afterwards only had a low-attendance. Regarding attendance barriers studies have found that; lack of support from leaders [8–11], training organized outside work hours [7], lack of information [12] and economic costs and transportation [7], negatively affected the attendance of voluntary participants in a WHPP. Previous studies have stressed that understanding the barriers that prevent participants from attending WHPPs are important to secure that the participants receive the full health benefits from the programme [7, 13–15]. Exploitation of the health potential of a WHPP requires deep insight into attendance barriers experienced by the participants who voluntarily sign-up for a WHPP but are somehow prevented from regularly participation.

Health care workers (HCWs) represent a high-risk population regarding musculoskeletal disorders. HCW are characterized as having a job; with high physical demands when handling patients, other manual work tasks with high peak force and many hours of walking and standing; as well as, working in awkward postures [16]. Female employees dominate this work group and they are also characterized with a high prevalence of being overweight, having low physical capacities and having a high prevalence of musculoskeletal pain [17]. Evidence suggests that it may be the combination of being overweight, having low physical capacity and high physical work demands that cause the high prevalence of musculoskeletal pain in HCWs [18–21]. Therefore, effective well-documented programmes for weight reduction, improving physical capacity and reducing musculoskeletal pain among HCWs are needed. It has been documented that increasing the physical capacity of people working in physically demanding professions, may lower the risk of long-term illness [22–24]. Therefore, programmes promoting health among HCWs can potentially reduce sickness and their subsequent absence from work. However, a successful programme requires knowledge on how to ensure HCWs participation these initiatives. The aim of the present study was to identify and explore attendance barriers which prevented the participation of female HCWs who voluntarly agreed to participte in a weekly WHPP. The exploration of the participants’ experiences may reveal factors that can be used to provide recommendations for implementation of future WHPP.

## Method

An inductive approach was used to obtain valid results for investigating WHPP attendance barriers [25, 26] and identifying new aspects of potential attendance barriers from empirical data. Data was generated through in-depth semi-structured qualitative interviews with female HCWs who voluntarily participated in the WHPP.

### Setting and design

The participants in this study were recruited from an on-going randomised controlled trial (RCT) named FRIDOM (FRamed Intervention to Decrease Occupational Muscle pain, NCT02843269, 06.27.2016 - retrospectively registered) [27]. FRIDOM was a collaboration between the University of Southern Denmark (SDU) and a Danish municipality. FRIDOM’s primary aim was to reduce neck and shoulder pain in female HCWs. Additionally, the study aimed to decrease sickness absenteeism, decrease sickness presenteeism, decrease lifestyle-diseases and to maintain the participants participation in regular physical exercise training one-year after the programme period. FRIDOM was tailored to a population of female HCWs who engaged in a WHPP. The WHPP included training in teams within work hours and consisted of a weekly one-hour multi-component programme including; 1) intelligent physical exercise training (IPET), dietary advice and weight loss (DAW) and cognitive behavioural training (CBT) [27]. The present study was a single-case study within the FRIDOM study and focused on the barriers participants experienced during the programme that prevented them from attending the weekly multi-component one-hour training sessions. In the present study, ‘attendance barriers’ are defined as factors that prevent attendance or negatively affect the participants’ ability to attend the weekly one-hour multi-component training session throughout the one-year programme period. In-depth semi-structured qualitative interviews with nine female HCWs who voluntarily participated in the WHPP took place in two home care workplaces and two retirement homes in Denmark.

### Participants and procedures

The FRIDOM participants were either female home care helpers or home care assistants (together named HCWs) who were working in the Geriatric health care sector in a Municipality in Jutland, Denmark. The HCWs were either working at a retirement home or were providing care to elderly citizens in their homes and had a shared office. For detailed in- and exclusion criteria in FRIDOM, please see Christensen et al. 2016 [27]. FRIDOM participants had varying ages, Body Mass Index (BMI) and attendance rates to the FRIDOM programme.

To be included in the present study, the participants had to be female and work as either a home care helper (a 14-month education) or home care assistant (a 21-month education, in addition to having a home care helper education). To explore any potential differences in organizational barriers, all participants from the two workplaces were asked to be part of the present study and 92% agreed to being interviewed if randomly selected. Because of the high consent rate to be interviewed we had no concern that the consenting interviewed participants would have different views than those who would not be interviewed. No incentives for agreeing to participate were offered. The two workplaces were chosen to avoid biased answers, since the person who carried out the interviews did not have any former contact with these workplaces. Next in the recruitment process, the participants who agreed to be interviewed were divided in two groups, those who were working in a retirement home and those who were providing home care in the citizens’ private homes. This division was important because of the difference in their work setting and work shifts (Table 1). Then, five from each of the two groups were randomly drawn to be interviewed. There were no significant differences between the participants in the present study and those who participated in the FRIDOM study in regards to age, BMI and attendance rate - Table 2. Furthermore, variation in the characteristics allowed for a broader understanding of the barriers experienced by the participants. On the day one of the interviews was to be conducted, one participant found it difficult finding time to participate due to being so busy at work and did not participate in the interview. The remaining nine participants were interviewed and as data saturation seemed to be reached within the nine interviews, we did not randomly draw for another participant - Table 1.

**Table 1:** Distribution of participants (n) in area, attendance rate and type of workplace

	Area	Attendance rate	Type of workplace
Participants, Syddjurs Municipality (9)	Hornslet (3)	High (2)	Home care (2)
		Low (1)	Retirement home (1)
	Rønde (6)	High (3)	Home care (1)
			Retirement home (2)
		Low (3)	Home care (3)

**Table 2:** Comparison of all FRIDOM and interviewed participants

	FRIDOM participants *N* = 348	Interviewed participants *N* = 9
Sex (%)		
Female	96.0	100.0
Age (Mean)	48.1	42.3
Educational background (%)		
Homecare helper	65.1	66.7
Homecare assistant	34.9	33.3
Workplace setting (%)		
Homecare	58.2	66.6
Retirement home	41.8	33.3
Working shift (%)		
Day	66.6	66.6
Evening	17.0	0.0
Night	1.2	11.1
Day/Evening	10.7	22.2
Evening/Night	22.5	0.0
Day/Evening/night	22.5	0.0
BMI (Mean)	27.8	26.4
BMI intervals^a^ (%):		
Underweight/normal range < 25	39.2	44.4
Overweight 25–29,9	28.3	33.3
Obesity > 30	32.5	22.2

(*BMI*) Body Mass Index

^a^ Intervals classified according to the WHO’s definition [26]

An interview guide using a retrospective perspective with open-ended questions was developed in order to explore attendance barriers experienced by the participants - see interview guide in Additional file 1. All participants were informed both verbally and in writing of the aim of the study before the interview. A female research assistant (JBL), who was experienced in conducting qualitative research, conducted all nine interviews. The research assistant had participated in the FRIDOM study as an instructor for some of the training sessions, but was not involved in any training sessions that these participants had attended. Therefore, the interviewer was familiar with the training concept, but the participants did not know the research assistant prior to the interviews. The face-to-face interviews were conducted in a quiet location at the participants’ workplace and lasted approximately 30 min. The interviews were audio recorded and transcribed by JBL in Microsoft Word, with participants’ identifiable information deleted from the transcripts. No specific software was used to carry out the analysis. The last author (JRC) supervised the research assistant throughout the data collection process for quality control.

### Analysis

Initially, JBL and JRC attained consensus on the procedure for the analysis of the data. Data driven analysis of the transcripts was used to obtain a condensed meaning of the transcript [25, 26]. Data analysis was conducted using the following five steps: 1) reading of the transcripts to provide an overview, 2) condensing the meaning of each interview to reduce the answers into sub-themes, 3) sub-themes from the interviews were gathered into broader main themes, 4) main themes were systematically linked to the aim of the study, 5) main themes were linked to the final description of the patterns in the data (Additional file 2: Coding categories). The inductive approach was chosen to explore the views of the participants and to avoid any pre-assumptions by using priori codes.

JBL and JRC analyzed one interview jointly. During this process, agreement on the content of sub-themes and main themes was attained. JBL completed all of the consecutive analysis of the interviews. The validation consisted of JRC reading through interviews, sub-themes and main themes. If JRC disagreed with the analysis, the discrepancies were discussed until a consensus was reached. See The COREQ Checklist in Additional file 3.

## Results

To illustrate the comparability of characteristics between the participants interviewed in this study to the participants in the FRIDOM study, see the overview in Table 2.

Specific characteristics of the interviewed participants are provided in Table 3. The participants were between 24 and 59 years of age, had a BMI between 18.8–34.7 and had a training attendance between 23 and 91%. They represented different work shifts and workplace settings.

**Table 3:** Characteristics of the participants (*N* = 9)

Participant number	Age range	Educational Background	Workplace Setting	Working Shift	Attendance %
P1	56–60	Health care assistant	Home care	Night	91.0
P2	31–35	Health care helper	Retirement home	Day	79.0
P3	31–35	Health care assistant	Retirement home	Day	84.0
P4	51–55	Health care helper	Home care	Day	23.0
P5	46–50	Health care assistant	Retirement home	Day	36.0
P6	51–55	Health care assistant	Home care	Day	75.0
P7	21–25	Health care helper	Home care	Day/Evening	81.0
P8	36–40	Health care helper	Home care	Day/Evening	23.0
P9	36–40	Health care helper	Home care	Day	46.0

### Experienced attendance barriers

The barriers experienced by the participants were grouped into six attendance barrier sub-themes and classified into three main themes: 1) *Organizational factors* (a. work inflexibility, b. lack of support from team leaders), 2) *Intervention factors* (c. training sessions organized outside normal work hours, d. incongruence between information received and what was experienced, e. content and intensity of the training sessions and 3) *Individual factors* (f. personal factors e.g. family, injury, pain, sickness). The number of participants who identified each sub-theme as an attendance barrier is shown in Table 4.

**Table 4:** Distribution of the three main themes and six sub-themes experienced by the participants

Main Themes	Sub-Themes (Attendance Barriers)	*N* = 9
Organizational factors	1. Work inflexibility	6
	2. Lack of support from team leaders	3
Intervention factors	3. Training session organized outside normal working hours	8
	4. Expectations not met between information received and reality	2
	5. Content and intensity of the program	4
Individual factors	6. Personal factors (e.g. family, injuries, pain, sickness)	7

#### Organizational factors

Due to the nature of the HCWs work, work duties had to be completed by their colleagues when the participants had to attend the WHPP. Having to burden their colleagues with their unfinished workload lead to feelings of guilt. One participant (P3) expressed:

*“You have to (stay at work) right? With your conscience… you have to stay at work…”* (P3)

Since the training sessions were organized within the participants’ work hours, the team leaders had to make sure that other HCWs could fill in while the participants attended the WHPP.

When a replacement was not available, the participants felt guilty and sometimes chose not to attend. Some participants said:

*“It is not machines we are working with, but humans. We can’t just leave. A colleague will have to take over…”* (P3)

*“It is probably your planning of the day when you are attending training (that is the biggest challenge).”* (P2)

Another sentiment mentioned by the participants as an attendance barrier, was having to work overtime on when certain job duties required immediate attention or had to be performed.

*“One of the times (not attending) was because a task needed to be done immediately. Another time was because a colleague was sick and I had to stay at work.”* (P5)

Furthermore, experiencing lack of mental resources or feelings of a high workload, could also be an attendance barrier.

*“It is hard working overtime, and I have a greater need for going home (than attending a training session).”* (P2)

Training session attendance was negatively affected if work-related activities, such as meetings and courses, were scheduled at the same time as the training session.

*“(non-attendance) had been because of attendance in a nightshift-meeting.”* (P1)

*“I have not been attending the last three weeks because I was attending a course.”* (P4)

Some participants experienced their team leaders having a negative attitude towards the WHPP. If their team leaders could not see the purpose of the WHPP and doubted its health promoting effects, it was seen as an attendance barrier.

*“Our leader hasn’t supported us as much as she could have, which sometimes negatively affected our enthusiasm for attending…”* (P2)

#### Intervention factors

If the WHPP was not organized within the participants’ work hours or in continuation with work, it was considered as an attendance barrier.

*“It can be one of the things (attendance barrier)… if you are not at the workplace working (when training sessions takes place).”* (P6)

Before the workplace started FRIDOM, information meetings were held to describe the project to the employees. Participants experienced incongruence of the information regarding the concept. It was not possible to schedule all training sessions during work hours as expected. Not fulfilling the expectations of the participants was experienced as irritating for one of the participants.

*“It means a lot to me to get what I was promised. It is called training within work hours. If it is not possible, then don’t call it that. It’s very irritating when people don’t keep their promises.”* (P9)

The WHPP was organized once a week and after work hours, which meant that the participants had to occasionally wait after work for the training program to begin. Eight out of the nine participants thought that when the WHPP was organized outside their work hours it was experienced as an attendance barrier. Due to the shift work, some participants had to attend training sessions on their day off.

*“It is very difficult to pull yourself together and attend (at the workplace) if you have the day off, because many of the training sessions take place on planned days off.”* (P3)

Proximity relative to the participants’ workplace and where they lived, was also experienced as an attendance barrier; since, some participants would need to incur extra time and economic costs due to the extra transportation.

*“It’s expensive… you know money… you know fuel costs.”* (P5)

Two participants expected that the content of the training sessions was going to be a wide variety of different sports and training activities.

*“Some had understood that it would be different activities. Not “just”* *here in the training room/gym. But you got the chance to try out some other sports.”* (P5)

Four of the HCWs expressed the content of the training sessions as an important factor for attending the training sessions.

*“It’s important what we do during the training session. If it is boring you don’t bother to show up... But I have heard some of the others would like to do more different activities.”* (P7)

Furthermore, if the intensity of the training session was experience as being above or below the physical capacity of the HCWs it was also identified as being an attendance barrier. One participant described the importance of the intensity of the training sessions.

*“… or if it doesn’t fit your physical capacity (it is an attendance barrier).”* (P7)

The HCWs acknowledged the difficulties the instructors had in balancing the content to meet the physical capacity of all the participants. The participants understood that the content of the training sessions was influenced by the competences of the instructors. The lack of fulfilling the participant’s expectations in previous training sessions may have had a negative effect on the participant’s future attendance.

*“When we are this many who are attending it is difficult (to meet expectations), because there must be something for everyone.”* (P9)

#### Individual factors

Low attendance was often due to individual factors. Participants sometimes prioritised their family over the training sessions if it was scheduled outside their work hours.

*“… I don’t want to spend less time with him (my child).”* (P9)

These participants expressed experiencing guilt if they had to spend time away from their loved ones in order to attend the WHPP outside work hours.

*“I’m not attending today because I need to visit my (sick) mother… Family comes first.”* (P2)

*“I feel guilty because they (my children) are in childcare/kindergarten for a longer time just because of my training.”* (P8)

If the participants experienced pain, injury or sickness; this was also identified as an attendance barrier.

*“I’ve injured my ankle. Sprained it and was on sick leave for a long time because of it.”* (P8)

## Discussion

The aim of the study was to explore the barriers that may prevent female HCWs from attending a weekly WHPP, and exploration of the participants’ experiences has the possibility to provide recommendations for successfully implementing future WHPPs. The participants’ experienced attendance barriers were categorized in three main themes, including six sub-themes: 1) *Organizational factors* (a. work inflexibility, b. lack of support from team leaders), 2) *Intervention factors* (c. training sessions organized outside normal work hours, d. incongruence between information received and what was experienced, e. content and intensity of the training sessions and 3) *Individual factors* (f. personal factors e.g. family, injury, pain, sickness). The most important factors seemed to be the *organizational factors* and *intervention factors*.

### Organizational factors

Three out of the nine participants experienced lack of support from team leaders as an attendance barrier. This may be related to the fact that the participants had shift work, which is a real challenge when organizing a WHPP. When team leaders did not support the WHPP, the time allowed for the participant to participate in the WHPP was not necessarily provided by the team leader. Six out of the nine participants stressed how experienced felling guilt when they left their colleagues or clients to attend the WHPP, which may hinder attendance. It is a challenge for team leaders to coordinate coverage for employees work shifts, if the employee has to be replaced by an extra staff member in order to attend the WHPP and may coincide with their available resources or lack thereof. The attendance barrier relating to the lack of support from leaders is well documented in the literature [9–11]. A study conducted in 2015 showed that even though the leaders were involved before starting a WHPP, the leaders were still the largest attendance barriers for employees [8]. When participants do not experience the acceptance from their leaders to prioritize a WHPP, this may result in absence from the programme [8]. Furthermore, attendance barriers can be affected if the team leaders do not believe in the health effects of the WHPP. Likewise, if the leaders do not feel ownership in the project, this too may negatively affect the attendance of the participants [8]. Thus, emphasizing the importance of securing the support from the team leaders when implementing a WHPP.

### Intervention factors

Nöhammer and colleagues identified training organized outside work hours as an attendance barrier, which is similar to the findings in the present study [7]. Eight out of the nine participants experienced an attendance barrier when the WHPP was not organized within work hours.

The present study showed that the intensity of the training content was an attendance barrier, which was supported by another study and thus underlines the importance of adjusting the training intensity to the employee’s physical capacities [7]. Moreover, offering training sessions with content in which all participants can participate is recommended [7]. However, the results of the present study showed that the participants also experienced loss of interest in the training sessions when the physical demands were too low and lead to a lower attendance. Therefore, it is important that the employees are well informed about the physical demands of the training sessions [7]. Providing correct information about the intervention decreases attendance barriers. The participants of the present study expressed that there were discrepancies in the information given before the intervention and during the intervention and these discrepancies were experienced as attendance barriers. One study found lack of information, poorly accessible information or information that was difficult to understand as an attendance barrier, which supports the present study’s findings [12]. The same study showed that when giving information, timing is essential to the interventions integrity and to the employees motivation for participating [12]. A stepped-wedge design was used in FRIDOM, which meant the participants were divided in three clusters and enrolled in the WHPP at different times. The design enabled all consenting employees to receive the intervention, superseded by a control group. Consequently, some participants received information about the WHPP 12-months before the actual intervention started. This might have influenced the participants’ motivation for attending. Likewise, if the discrepancies they experienced between the information they remembered and the actual programme they experienced, may have increased over time.

In order to reduce WHPP attendance barriers, it is important to clearly state information to the participants about the intensity and content of the training, before the programme is scheduled to start and again at the start of the programme.

### Individual factors

The WHPPs one-hour weekly sessions were aimed to be held within the participants work hours. However, the present study showed that this was not always possible, due to the varying shifts of the HCWs work. Training outside of work hours was regarded as an attendance barrier for the participants, which resulted in a negative effect on programme attendance. Several studies have found that individual factors can be an attendance barrier in WHPPs [7, 8, 28]. Nöhammer and colleagues found that economic costs and transportation time affected training attendance and other health initiatives at the workplace [7]. Prioritising family instead of training was not identified as an attendance barrier in a review by Robroek and colleagues [6]. The review included studies with different populations (*N* = 53.059); e.g., university employees, insurance company employees and health care system employees [6]. The review revealed that employees who were married or living with a partner had more frequently attendance because of the support their family-members provided, compared to employees who were single [6]. This is contradicted in the present study that found prioritizing ones family negatively affected the attendance rate. The participants did not mention support from family-members, but explained they experienced feelings of guilt for attending the WHPP instead of spending time with their family-members. Furthermore, if participants experience physical injuries or sickness in may be difficult to optimize their attendance rate.

Finding the most optimal time for the intervention to take place may facilitate a higher attendance rate. For employees who have shift work require planned has to be done thoroughly in order to enable their participation in training sessions.

### Strength and limitations

The present study focused on the understudied aspect of attendance barriers to participation in WHPP initiatives and the inductive approach provided new insight into attendance barriers and how they hindered the attendance of participants who wanted to attend. The recruitment systematically selected all participants from two specific workplaces and then the participants were divided into two groups; a group who were working at a retirement home and a group who were providing home care in citizens’ private homes. Then, five participants from each group were randomly drawn to be interviewed. After the 10 participants were drawn, it was discovered that one participant had changed their workplace from a retirement home to a home care setting, which resulted in having six participants in the home care group and four participants in the retirement home group. Following the selection process, the participants were randomly recruited, with a aim that the sample would include participants with different characteristics in age, BMI and attendance rate, which was obtained. If the sample did not show a variety, the plan was to continue randomly draw one participant at a time until a certain variation was achieved.

However, the study has low external validity since it was only female HCWs who participated. Therefore, the results cannot be considered applicable for male HCWs or females and males with other jobs or higher education levels. Despite a smaller sample size, data saturation seemed to be reached with no new themes emerging in the last interviews.

FRIDOM is a research project including tests and questionnaires, which demands more of the participants then an ordinary WHPP. This might have influenced the participants’ motivation for not-attending the training sessions.

This study only identified attendance barriers identified by participants who volunteered to participate in a WHPP, since the study was interested in finding reasons for low attendance rates despite the participants stating that they really wanted to attend. Although more than 85% volunteered to participate in the FRIDOM project, future research may also explore the barriers for not enrolling in a WHPP, which may overlap with the findings of participants with low attendance rates in the present study.

## Conclusion

Important factors for minimising attendance barriers in future WHPPs are primarily organizational and intervention factors. Securing the support of the team leaders was essential when implementing a WHPP, as the team leaders’ support might decrease the feeling of guilt experienced by participants when attending the WHPP during work hours. The team leaders planning of employees-work shifts are of great importance when dealing with the contextual challenges of working in the health care sector. It is important for the future implementation of similar programmes to ensure the possibility of attending the WHPP during work hours or in continuation of work time. By carefully planning when the training sessions are to be held this can reduce some individual barriers - so participants do not have to prioritize training over their family. If it is not possible to ensure that the WHPP will only take place within work hours, this should be clarified to the potential participants before the start of the programme. Thereby, allowing the participants the opportunity to decide whether to prioritize the potential health benefits before participating in the intervention. The participants’ expectations of the programme should be clearly understood before agreeing to participate. Also, it is an advantage to involve the employees participating when deciding the content and intensity of the intervention and continually doing so, throughout the programme. Finally, future WHPP with similar stepped-wedge design should offer an extra information meeting with each group just before the programme begins.

To summarize, in order to increase attendance rates when offering a WHPP it is important to carefully consider relevant organizational, intervention, and individual factors.

## Additional files

Additional file 1: Interview guide. Information’s given to the informants before the interviews and the interview guide that was followed. (DOCX 75 kb)

Additional file 2: Coding categories. An overview of the procedure for the analysis of the data. Presenting an overview of how the identified attendance barriers were reduced into sub-themes and further gathered into broader main themes. (DOCX 19 kb)

Additional file 3: The COREQ Checklist. The COREQ Checklist with comments filled out. (DOCX 24 kb)

## Abbreviations

BMI: Body Mass Index
FRIDOM: FRamed Intervention to Decrease Occupational Muscle pain
HCWs: Health care workers
RCT: Randomised controlled trial
WHPP: Workplace Health Promotion Programme
