# Supplementary material for: Attendance barriers experienced by female health care workers voluntarily participating in a multi-component health promotion programme at the workplace
Source: BMC Public Health. 2018 Dec 4;18:1340. doi: 10.1186/s12889-018-6254-3 (PMC6278076; doi:10.1186/s12889-018-6254-3)
Supplement: Supplementary file 1 — Interview guide. Information’s given to the informants before the interviews and the interview guide that was followed. (DOCX 75 kb) [file 12889_2018_6254_MOESM1_ESM.docx]

**Additional file 1. Interview guide**

INTRODUCTION TO THE INFORMANTS
"From the FRIDOM teams we have experienced that there are many who only come once in a while or, for longer periods do not come to the weekly team training. It also applies to those who say it has been good to attend the training teams every time they have been there and that they really want to come. We therefore investigate the reasons that make people attend to the training and what hindres them to attend. By doing this investigation, we hope to be able to increase attendance rates in future projects, so that the benefits from the exercise training will be the greatest possible for all.
The interview is expected to last approx. 20 minutes and data processed anonymously".

INTERVIEW GUIDE

1. What do you think about the training session? How do you experience the attendance in the training team? Can you recognize our statement?
2. What challenges did you experience in order to attend the weekly training sessions?
3. If you could not attend, what was the reason preventing you from attending the training sessions?
4. What motivated you to attend the training session at the workplace?
5. Did you experience any challenges?
6. What are your thoughts about the training that took place at the workplace?
7. Did you experience any challenges about the training being at the workplace?
8. What areas can be improved to ensure higher participation in the training session?
9. Is there something that could be done differently in general to increase the attendance in relation to FRIDOM?
