# Supplementary material for: Attendance barriers experienced by female health care workers voluntarily participating in a multi-component health promotion programme at the workplace
Source: BMC Public Health. 2018 Dec 4;18:1340. doi: 10.1186/s12889-018-6254-3 (PMC6278076; doi:10.1186/s12889-018-6254-3)
Supplement: Supplementary file 2 — Coding categories. An overview of the procedure for the analysis of the data. Presenting an overview of how the identified attendance barriers were reduced into sub-themes and further gathered into broader main themes. (DOCX 19 kb) [file 12889_2018_6254_MOESM2_ESM.docx]

**Additional file 2: Coding categories**

| **Attendance barriers** | **Sub-themes** | **Main theme** |
| --- | --- | --- |
| Working conditions | Inflexibility at work | Organizational factors |
| Feeling guilty when leaving colleagues with their work load | Inflexibility at work | Organizational factors |
| Leaving colleagues with extra work | Inflexibility at work | Organizational factors |
| Difficult to leave work to attend training | Inflexibility at work | Organizational factors |
| Team leaders do not believe in the health benefits of the intervention | Lack of support from team leaders | Organizational factors |
| Lack of support from team leaders | Lack of support from team leaders | Organizational factors |
| The time the training sessions are scheduled | Training organized outside work hours | Intervention factors  Organizational factors |
| Training sessions scheduled on a day off from work | Training organized outside work hours | Intervention factors  Organizational factors |
| Waiting time between work and the training session | Training organized outside work hours | Intervention factors  Organizational factors |
| Information expectations | Incongruent information | Intervention factors |
| Lack of dissemination of results to leaders | Implementation of the project | Intervention factors |
| Intensity too low/ not physically challenging | Intensity and content of the training session | Intervention factors |
| To much socializing at the training session and not enough training (sedentary) | Intensity and content of the training session | Intervention factors |
| Physical demands (intensity of training) | Intensity and content of the training session | Intervention factors |
| Prioritize other activities on the day off instead of training | Training outside working hours | Individual factors |
| Physical injuries | Personal factors | Individual factors |
| Prioritizing spending time with their family | Personal factors | Individual factors |
| Family demands (e.g. children, husband) | Personal factors | Individual factors |
| Long transportation to the workplace (time and costs) | Personal factors | Individual factors |
| Priority | Personal factors | Individual factors |
| Pain | Personal factors | Individual factors |
| Stress | Personal factors | Individual factors |
| Sickness | Personal factors | Individual factors |
