# Supplementary material for: Attendance barriers experienced by female health care workers voluntarily participating in a multi-component health promotion programme at the workplace
Source: BMC Public Health. 2018 Dec 4;18:1340. doi: 10.1186/s12889-018-6254-3 (PMC6278076; doi:10.1186/s12889-018-6254-3)
Supplement: Supplementary file 3 — The COREQ Checklist. The COREQ Checklist with comments filled out. (DOCX 24 kb) [file 12889_2018_6254_MOESM3_ESM.docx]

**Additional file 3.** **The** **COREQ Checklist**

| **No** | **Item** | **Guide question/description** | **Comments** |
| --- | --- | --- | --- |
| **Domain 1: Research team and reflexivity** |  |  |  |
| Personal Characteristics |  |  |  |
| 1. | Interviewer/facilitator | Which author/s conducted the interview or focus group? | Jonna Benner Lundgaard (JBL) |
| 2. | Credentials | What were the researcher's credentials? *E.g. PhD, MD* | Bachelor of Science in Sports Science |
| 3. | Occupation | What was their occupation at the time of the study? | Master of Science in Sports Science (student) |
| 4. | Gender | Was the researcher male or female? | Female |
| 5. | Experience and training | What experience or training did the researcher have? | Training at the university |
| Relationship with participants |  |  |  |
| 6. | Relationship established | Was a relationship established prior to study commencement? | No |
| 7. | Participant knowledge of the interviewer | What did the participants know about the researcher? e*.g. personal goals, reasons for doing the research* | The researchers reasons for doing the research |
| 8. | Interviewer characteristics | What characteristics were reported about the interviewer/facilitator? e.g. *Bias, assumptions, reasons and interests in the research topic* | JBL has instructed some of the training sessions in relation to FRIDOM but not the sessions the interview participants attended |
| **Domain 2: study design** |  |  |  |
| Theoretical framework |  |  |  |
| 9. | Methodological orientation and Theory | What methodological orientation was stated to underpin the study? *e.g. grounded theory, discourse analysis, ethnography, phenomenology, content analysis* | Content analysis  Method: Inductive approach  Design: single-case study |
| Participant selection |  |  |  |
| 10. | Sampling | How were participants selected? *e.g. purposive, convenience, consecutive, snowball* | Purposive random sampling |
| 11. | Method of approach | How were participants approached? e*.g. face-to-face, telephone, mail, email* | Face-to-face |
| 12. | Sample size | How many participants were in the study? | Nine |
| 13. | Non-participation | How many people refused to participate or dropped out? Reasons? | One participant had difficulty finding time to participate due to time constraints |
| Setting |  |  |  |
| 14. | Setting of data collection | Where was the data collected? e*.g. home, clinic, workplace* | Workplace |
| 15. | Presence of non-participants | Was anyone else present besides the participants and researchers? | No |
| 16. | Description of sample | What are the important characteristics of the sample? *e.g. demographic data, date* | Age, gender, education, workplace, type of shifts (day, evening or night), degree of attendance |
| Data collection |  |  |  |
| 17. | Interview guide | Were questions, prompts, guides provided by the authors? Was it pilot tested? | Please see “Appendix: Interview guide”  No pilot test was performed |
| 18. | Repeat interviews | Were repeat interviews carried out? If yes, how many? | No |
| 19. | Audio/visual recording | Did the research use audio or visual recording to collect the data? | Audio recording |
| 20. | Field notes | Were field notes made during and/or after the interview or focus group? | No |
| 21. | Duration | What was the duration of the interviews or focus group? | Approximately 30 minutes |
| 22. | Data saturation | Was data saturation discussed? | Yes, please see original article |
| 23. | Transcripts returned | Were transcripts returned to participants for comment and/or correction? | No |
| **Domain 3: analysis and findings** |  |  |  |
| Data analysis |  |  |  |
| 24. | Number of data coders | How many data coders coded the data? | One (JBL) |
| 25. | Description of the coding tree | Did authors provide a description of the coding tree? | Yes, please see “Appendix: Coding categories” |
| 26. | Derivation of themes | Were themes identified in advance or derived from the data? | Three main themes emerged from the data |
| 27. | Software | What software, if applicable, was used to manage the data? | Microsoft Word |
| 28. | Participant checking | Did participants provide feedback on the findings? | No |
| Reporting |  |  |  |
| 29. | Quotations presented | Were participant quotations presented to illustrate the themes / findings? Was each quotation identified? e*.g. participant number* | Yes  Yes |
| 30. | Data and findings consistent | Was there consistency between the data presented and the findings? | Yes |
| 31. | Clarity of major themes | Were major themes clearly presented in the findings? | Yes |
| 32. | Clarity of minor themes | Is there a description of diverse cases or discussion of minor themes? | Not relevant |
